# Clinical Relevance of the serum CTLA-4 in Cats with Mammary Carcinoma

**DOI:** 10.1038/s41598-020-60860-3

**Published:** 2020-03-02

**Authors:** Ana Catarina Urbano, Catarina Nascimento, Maria Soares, Jorge Correia, Fernando Ferreira

**Affiliations:** 1 CIISA - Centro de Investigação Interdisciplinar em Sanidade Animal, Faculdade de Medicina Veterinária, Universidade de Lisboa, Avenida da Universidade Técnica, Lisboa, 1300-477 Portugal; 2 Research Center for Biosciences and Health Technologies (CBiOS), Faculdade de Medicina Veterinária, Universidade Lusófona de Humanidades e Tecnologias (ULHT), Lisboa, 1749-024 Portugal

**Keywords:** Cancer, Biomarkers, Oncology

## Abstract

Cytotoxic T lymphocyte associated antigen 4 (CTLA-4) serves an important role in breast cancer progression, which has led to the development of novel immunotherapies aimed at blocking tumor immune evasion. Although feline mammary carcinoma is increasingly recognized as a valuable cancer model, no studies on CTLA-4 function had been conducted in this species. The serum CTLA-4, TNF-α and IL-6 levels of 57 female cats with mammary carcinoma were determined by ELISA, and immunohistochemistry was performed to evaluate CTLA-4 and FoxP3 expression in tumor cells and interstitial lymphocytes. The results obtained show that serum CTLA-4 levels are increased in cats with mammary carcinoma (*P* = 0.022), showing an association with a number of clinicopathological features: smaller tumor size, *P* < 0.001; absence of tumor necrosis, *P* < 0.001; non-basal status, *P* < 0.02 and HER-2-positive status. Additionally, a strong positive correlation was found between serum CTLA-4 levels and serum TNF-α (*R* = 0.88, *P* < 0.001) and IL-6 levels (*R* = 0.72, *P* < 0.001). Concerning the CTLA-4 and FoxP3 expression, although detected in both interstitial lymphocytes and tumor cells, a positive association was found only between interstitial CTLA-4 and FoxP3 expressions (*R* = 0.387, *P* = 0.01), which is negatively associated with the serum CTLA-4 levels (*P* = 0.03). These findings provide a preliminary step in the characterization of immune profiles in feline mammary carcinoma, uncovering a molecular rationale for targeted therapy with CTLA-4 pathway inhibitors. Finally, by strengthening the hypothesis of an immunomodulatory role for this regulator, we further validate the utility of spontaneous feline mammary carcinoma as a model for human breast cancer.

## Introduction

Although the dog has been the focus in comparative oncology, cats also have clear benefits over rodent laboratory models of cancer, namely, they are immunocompetent and share the same environment as humans, reflecting more accurately the complex interplay between genetics and environmental risk factors, as well as the role of the immune system and tumor microenvironment (TME). Furthermore, the higher similarity between the cat and human genomes, together with the increased frequency of several tumor types (e.g. injection-site sarcoma, oral squamous cell carcinoma, lymphoma and malignant mammary tumors)^1–3^, anticipate that the cat may be a superior model. Feline mammary carcinoma shares many epidemiological and histopathological characteristics with human breast cancer, in particular, the human epidermal receptor-2 (HER-2) positive and triple negative (TN) subtypes, having been proposed as a suitable model for their study^4–6^, and offers further opportunities for studying certain aspects of tumor biology, such as the crosstalk between the immune system and tumor development.

Chronic inflammation is a well-established risk factor for several cancers, with lymphocytes playing a pivotal role in the development of chronic inflammatory conditions^7^. The activation of T-lymphocytes requires two signals: after recognition of the MHC-peptide complex by the T-lymphocyte receptor (TCR), a second signal is provided by the binding of the cluster of differentiation 28 (CD28) to its ligands CD80/CD86 on antigen presenting cells (APCs). This interaction leads to Cytotoxic T-lymphocyte associated protein 4 (CTLA-4, CD152) translocation to the cell surface. CTLA-4 is an immunoglobulin superfamily cell adhesion molecule, exclusively expressed on lymphocytes, and a homologue of CD28. Because CTLA-4 has higher affinity for CD80/CD86 it can interrupt the activation signal delivered by CD28 and deliver its own signal which downregulates T-lymphocyte function^8^, triggering a negative feedback loop that is essential to the maintenance of immune self-tolerance and homeostasis. Feline CTLA-4 shows a high homology with the human and murine counterparts (86.6% and 76.2% respectively), presenting an hexapeptide motif (MYPPPY) within the extracellular domain responsible for interaction with the B7 ligands that is highly conserved among mammals^9^.

In humans, CTLA-4 also exists as a soluble isoform (sCTLA-4) in sera, an alternative splicing product of the CTLA-4 gene, mainly expressed on resting T-lymphocytes cells or at the post-activated state^10^. Elevated sCTLA-4 levels have been reported in several cancers, including esophageal^11^, lung^12^, and breast carcinoma^13^, however, the clinical implications of CTLA-4 in the tumor microenvironment are still controversial. While it has been demonstrated that CTLA-4 expression can be an important mechanism of tumor immune evasion^14,15^, with high levels of CTLA-4 expression in tumor tissues being associated with poor overall survival^16^, a recent study on breast cancer patients found that elevated sCTLA-4 levels were associated with improved survival^17^ and several other studies have showed significant correlations between CTLA-4 and improved OS in other cancer types^12^.

Since sCTLA-4 has also been found to be elevated in several inflammatory human disorders, where it’s able to display functional activities, acting on the proliferation capability of T-lymphocytes and modulating the secretion of cytokines^18^, we hypothesized that the elevated levels of sCTLA-4 in breast cancer might be a reflection of crosstalk between immune cells and the TME. Our investigation, therefore, studied the relationship between serum CTLA-4 and standard inflammatory markers, namely the pro-inflammatory cytokines, TNF-α and IL-6, which have emerged as central players linking inflammation and cancer. To further investigate this crosstalk, we evaluated the expression of CTLA-4 and FoxP3, an immune cell marker for regulatory T-lymphocytes (Tregs), in the TME. Tregs are a subset of CD4+ T-lymphocytes, known to highly infiltrate various tumor types in both humans and felines^19^, that, unlike other T-lymphocyte subsets, express CTLA-4 constitutively, and seem to be the primary source of sCTLA-4^20^.

## Results

### Cats with mammary carcinoma show increased serum CTLA-4 levels being related with small tumor size, absence of tumor necrosis, non-basal status and HER-2 positive status

**Figure 1:**
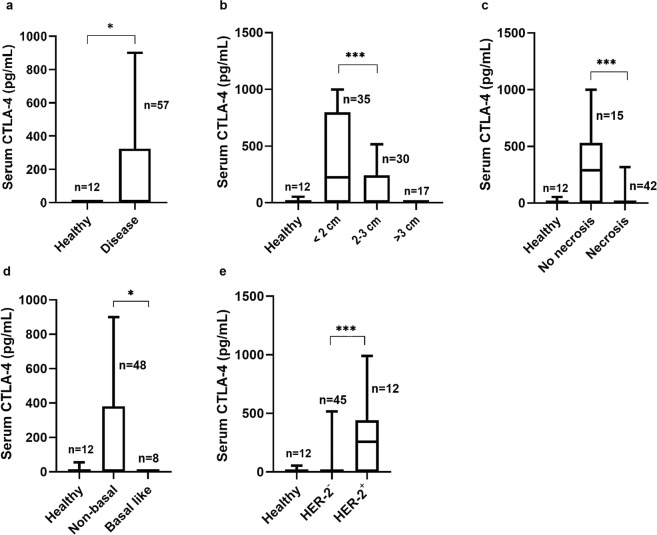
Box plot analysis of serum cytotoxic T-lymphocyte associated protein 4 (CTLA-4) levels in cats with mammary carcinoma and their association with clinicopathological and immunohistochemical parameters: (**a**) disease status (**b**) tumor size (cm); (**c**) tumor necrosis; (**d**) basal status; (**e**) HER-2 status. *P < 0.05; **P < 0.001; ***P < 0.0001.

Serum CTLA-4 levels were detected in 23 of the 54 cats with mammary carcinoma (43%), showing a median of 459.4 pg/mL (range 77–999.3 pg/mL), contrasting with undetectable levels in all healthy animals (detection limit = 31.3 pg/mL). As such, serum CTLA-4 levels in the cancer group were significantly higher than those in the healthy group (*P* = 0.022; Fig. 1a). Further statistical analysis showed that higher serum CTLA-4 levels are associated with small tumor size (*P* < 0.001; Fig. 1b), absence of tumor necrosis (*P* < 0.001; Fig. 1c), and non-basal status (*P* = 0.002; Fig. 1d). Serum CTLA-4 levels were also significantly increased in cats with mammary carcinomas immunohistochemically classified as HER-2-positive (*P* < 0.001; Fig. 1e).

### Serum CTLA-4 levels are correlated with serum TNF-α and IL-6 levels

**Figure 2:**
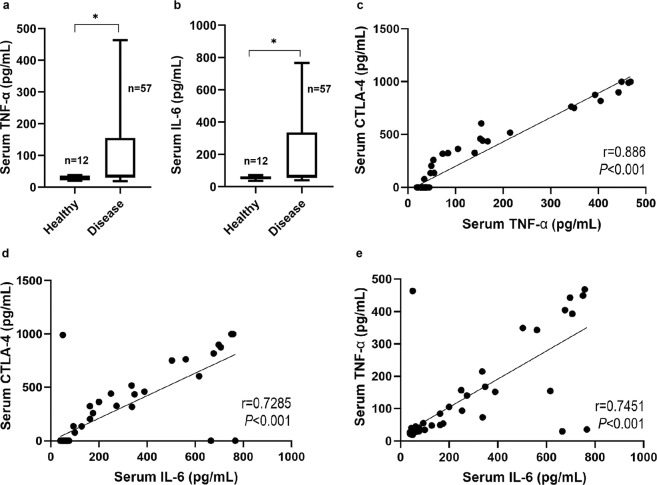
Box plot analysis of serum cytokine levels and Pearson correlation with serum cytotoxic T-lymphocyte associated protein 4 (CTLA-4): (**a**) tumor necrosis factor alpha (TNF-α) serum levels; (**b**) Interleukin 6 (IL-6) serum levels; (**c**) correlation between serum CTLA-4 and TNF-α (R = 0.8860, P < 0.001); (**d**) correlation between serum CTLA-4 and IL-6 (R = 0.7285, P < 0.001); (**e**) correlation between TNF-α and IL-6 (R = 0.7451, P < 0.001). *P < 0.05; **P < 0.001; ***P < 0.0001.

Serum TNF-α and IL-6 levels were significantly higher in cats with mammary carcinoma compared to healthy controls (*P* = 0.011 and *P* = 0.021 respectively; Fig. 2a,b), being detectable in all cats with mammary carcinoma enrolled in the study, showing a median of 36.10 pg/mL (range 19.11–463.5 pg/mL) and 65.94 pg/mL (range 39.17–766.5 pg/mL), respectively. As previously reported, cytokine levels were also detectable in healthy animals, showing a median of 32.79 pg/mL (range 20.36–37.5 pg/mL) and 56.48 pg/mL (range 36.09–71.07 pg/mL), respectively. In addition, a strong positive correlation was found between serum CTLA-4 and both serum TNF-α (*R* = 0.8860, *P* < 0.001; Fig. 2c) and IL-6 levels (*R* = 0.7285, *P* < 0.001; Fig. 2d), and also between serum TNF-α and IL-6 levels (*R* = 0.7451, *P* < 0.001; Fig. 2e).

### CTLA-4 is expressed in interstitial lymphocytes being negatively correlated with serum CTLA-4 levels in cats with mammary carcinoma

**Figure 3:**
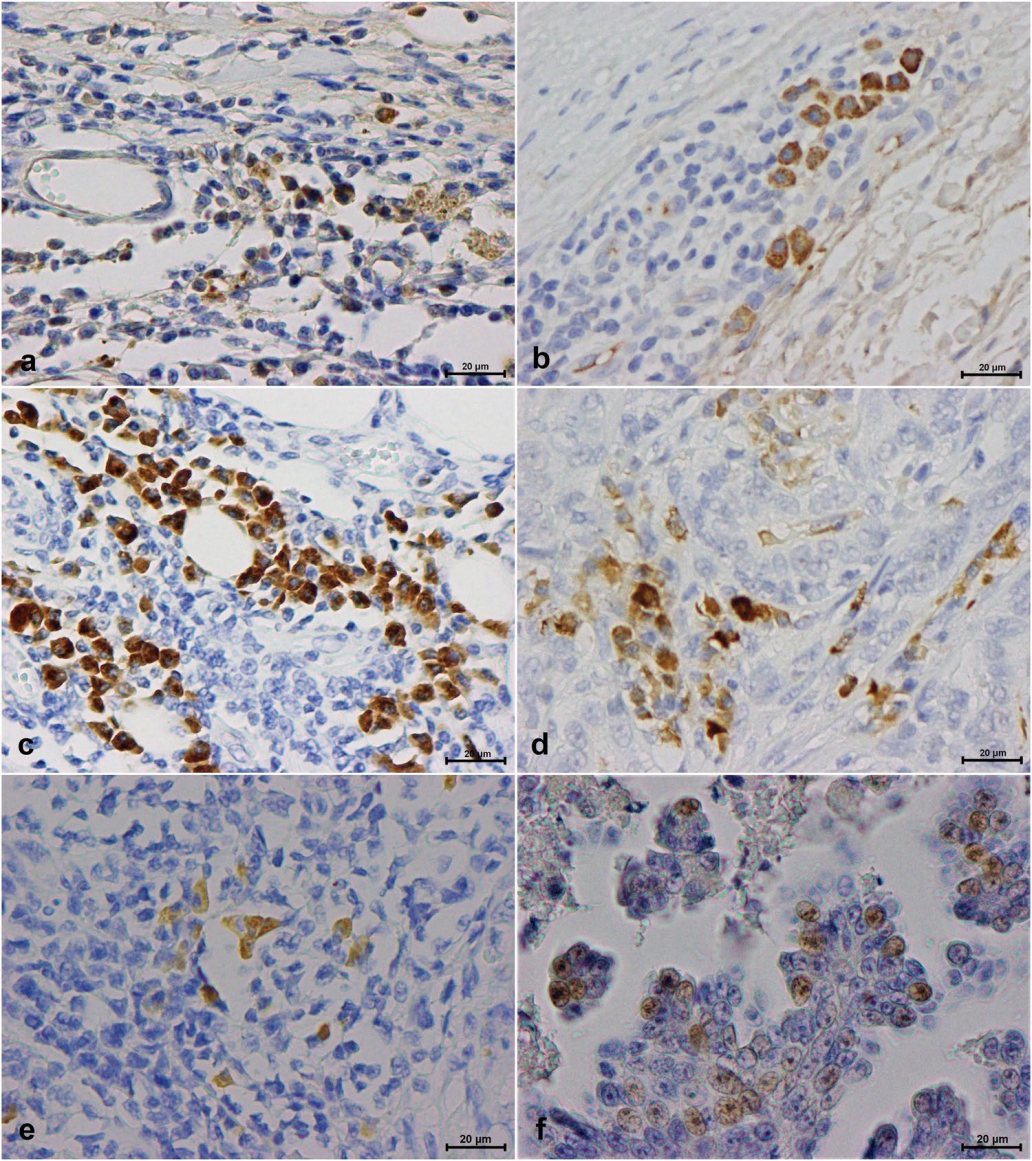
Serum cytotoxic T-lymphocyte associated protein 4 (CTLA-4) and Forkhead box (Fox)P3 expression in interstitial lymphocytes and tumor tissue: (**a**) CTLA-4 intensity 1+, showing a low number of reactive cytoplasmic granules (from #21; Table 1); (**b**) CTLA-4 intensity 2+, showing a moderate number of reactive cytoplasmic granules (from #26; Table 1); (**c**) CTLA-4 intensity 3+, showing a high number of reactive granules packed in the cytoplasm (from #28; Table 1); (**d)** CTLA-4 expression in tumor tissue; (**e**) FoxP3 interstitial expression; (**f**) FoxP3 expression in tumor tissue (40x objective).

**Figure 4:**
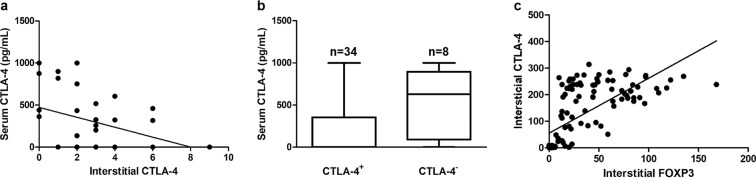
Associations of serum cytotoxic T-lymphocyte associated protein 4 (CTLA-4) levels with interstitial CTLA-4 and FoxP3 expression: (**a**) Spearman rank test of CTLA-4 serum levels and CTLA-4 interstitial expression; (**b**) Mann-Whitney U test of CTLA-4 serum levels and CTLA-4 interstitial expression; (**c**) Pearson correlation of CTLA-4 serum levels and FoxP3 interstitial expression.

Immunostaining analysis revealed that from the 49 tumor specimens evaluated, 34 (81%) showed a CTLA-4 positive expression in interstitial lymphocytes and 2 (4%) in tumor cells (Table 1). While in interstitial lymphocytes, CTLA-4 expression was detected in the cell membrane and cytoplasm, in tumor cells, the staining was exclusively cytoplasmic. Both interstitial lymphocytes and tumor cells showed a granular staining pattern of differing intensities (Fig. 3). A significant negative correlation was found between serum CTLA-4 levels and CTLA-4 expression in interstitial lymphocytes (*R* = −0.3425, *P* = 0.03) (Fig. 4a). To further confirm this association, tumors were divided into CTLA-4-positive and CTLA-4-negative groups, according to the expression of CTLA-4 in interstitial lymphocytes. The strong negative association between the serum levels and interstitial CTLA-4 expression was reconfirmed by the Mann-Whitney U test (*P* = 0.03) (Fig. 4b). No CTLA-4 expression was detected in the normal control tissue (data not shown).

**Table 1:** Evaluation of CTLA-4 positivity in interstitial lymphocytes showing percentages of positive cells (+Cells), intensity of the staining reactions, IHC scores, interpretation, and percentages of stained tumor cells (TC).

No	+Cells (%)	Intensity	Score	Interpretation	+TC (%)
1	0	0	0	Negative	0
2	5	2+	2	Positive	0
3	2	2+	2	Positive	0
4	5	3+	3	Positive	0
5	0	0	0	Negative	0
6	10	3+	6	Positive	0
7	5	3+	3	Positive	0
8	0	0	0	Negative	0
9	5	3+	3	Positive	0
10	5	3+	3	Positive	0
11	1	2+	2	Positive	0
12	15	2+	4	Positive	0
13	5	2+	2	Positive	0
14	0	0	0	Negative	0
15	2	2+	2	Positive	0
16	NS	NS	ND	ND	0
17	NS	NS	ND	ND	0
18	NS	NS	ND	ND	0
19	NS	NS	ND	ND	0
20	2	2+	2	Positive	0
21	3	1+	1	Negative	0
22	3	3+	3	Positive	0
23	NS	NS	ND	ND	0
24	2	2+	2	Positive	0
25	1	1+	1	Negative	0
26	20	2+	4	Positive	0
27	30	3+	6	Positive	0
28	20	3+	6	Positive	0
29	10	1+	2	Positive	0
30	NS	NS	ND	ND	0
31	1	1+	1	Negative	0
32	20	2+	4	Positive	1
33	1	1+	1	Negative	0
34	20	3+	6	Positive	0
35	40	2+	6	Positive	0
36	30	3+	6	Positive	0
37	1	2+	2	Positive	0
38	2	2+	2	Positive	0
39	2	2+	2	Positive	0
40	40	3+	9	Positive	0
41	NS	NS	ND	ND	0
42	50	2+	6	Positive	0
43	30	3+	6	Positive	3
44	15	2+	4	Positive	0
45	10	2+	4	Positive	0
46	40	3+	9	Positive	0
47	10	3+	6	Positive	0
48	3	3+	3	Positive	0
49	5	2+	2	Positive	0

NS – none seen; ND – not determined.

### FoxP3 and CTLA-4 expression is correlated in interstitial lymphocytes

From the 49 tumor specimens, 35 (74%) and 15 (31%) were scored as FoxP3-positive in interstitial lymphocytes and in tumor cells, respectively (Table 2). Whereas in interstitial lymphocytes, most of FoxP3 staining was detected in the cytoplasm with some cells exhibiting nuclear expression (Fig. 3e), only discrete FoxP3^+^ dots were found in the nucleus of tumor cells (Fig. 3f). Although, no significant correlation was observed between serum CTLA-4 levels and FoxP3 expression in either interstitial lymphocytes or tumor cells, FoxP3 expression in interstitial lymphocytes was strongly correlated with the CTLA-4 IHC score of interstitial lymphocytes (*R* = 0.387, *P* = 0.01, Fig. 4c). No FoxP3 expression was detected in the normal control tissue (data not shown).

**Table 2:** Evaluation of FoxP3 positivity in interstitial lymphocytes showing the percentages of positive cells (+Cells), intensity of the staining reactions, IHC scores, interpretation, and percentages of stained tumor cells (TC).

No	+Cells (%)	Intensity	Score	Interpretation	+TC (%)
1	5	2+	2	Positive	0
2	1	2+	2	Positive	1
3	5	3+	3	Positive	0
4	30	3+	6	Positive	0
5	1	2+	2	Positive	1
6	1	2+	2	Positive	0
7	3	3+	3	Positive	0
8	1	2+	2	Positive	1
9	1	2+	2	Negative	3
10	4	3+	3	Positive	0
11	2	2+	2	Positive	0
12	20	2+	4	Positive	0
13	5	2+	2	Positive	3
14	2	1+	1	Negative	0
15	3	3+	3	Positive	0
16	5	3+	3	Positive	3
17	1	3+	3	Positive	0
18	10	3+	6	Positive	0
19	1	2+	2	Positive	5
20	1	2+	2	Positive	0
21	10	1+	2	Positive	10
22	10	2+	4	Positive	10
23	0	0	0	Negative	0
24	30	2+	4	Positive	1
25	1	1+	2	Positive	0
26	20	2+	4	Positive	0
27	20	2+	4	Positive	0
28	10	3+	6	Positive	1
29	1	1+	1	Negative	1
30	5	2+	2	Positive	1
31	1	1+	1	Negative	0
32	1	2+	2	Positive	0
33	0	0	0	Negative	0
34	10	3+	6	Positive	0
35	0	0	0	Negative	0
36	15	3+	6	Positive	1
37	0	0	0	Negative	0
38	1	2+	2	Positive	0
39	0	0	0	Negative	0
40	0	0	0	Negative	1
41	0	0	0	Negative	0
42	3	2+	2	Positive	0
43	3	3+	3	Positive	0
44	NS	NS	ND	ND	0
45	1	2+	2	Positive	0
46	2	3+	3	Positive	0
47	1	3+	3	Positive	0
48	0	0	0	Negative	0
49	NS	NS	ND	ND	0

NS – none seen; ND – not determined.

## Discussion

Cats are increasingly being used as models for the study of spontaneous human diseases, and the epidemiological and histological similarities between feline mammary tumors and human breast cancer make this species a valuable cancer model for comparative oncology studies. To the best of our knowledge, the present study was the first to evaluate the serum CTLA-4 profiles and the serum TNF-α and IL-6 levels in cats with mammary carcinoma. The results presented clearly demonstrate that serum CTLA-4 levels are increased in cats with mammary carcinoma, in accordance with previous results reported in human malignant tumors^11–13^. This event may relate to an ongoing inflammatory response in the tumor microenvironment, where post-activated T-lymphocytes are actively expressing sCTLA-4. In parallel, the proteolytic cleavage of the full length isoform (mCTLA-4) concurrent with T-lymphocyte exhaustion, a progressive loss of effector function due to prolonged antigen stimulation observed in cancer patients, may also be contributing factors^21^. Results obtained also demonstrated that higher serum CTLA-4 levels are correlated with a number of clinicopathological features (i.e. smaller tumors, absence of necrosis, HER2-positive status, and non-basal status). Although these findings seem to be counterintuitive as increasing concentrations of CTLA-4 should inhibit T-lymphocyte activity, some studies have suggested that, while in the resting T-lymphocytes only sCTLA-4 is expressed, inhibiting CD28-ligand interactions, in later tumor phases, mCTLA-4 is overexpressed, with sCTLA-4 interfering with mCTLA-4-ligand interactions, thus enhancing T-lymphocyte reactivity by preventing the transduction of inhibitory signals^18,22,23^. It has also been suggested that CTLA-4 can mediate negative signals into tumor cells, comparable to those observed in resting T-lymphocytes, thus inhibiting tumor cell proliferation and/or inducing apoptotic cell death^24^. In this context, the association found between elevated serum CTLA-4 and HER2-positive tumors would seem paradoxical, as this subtype is frequently associated with more aggressive features and poorer prognosis in humans. However, a recent study reported a positive correlation between higher HER2 mRNA levels and better clinical outcome in feline mammary carcinoma^25^, while a study using a mouse model of HER2 positive breast cancer found that induced intratumoral expression of a CTLA-4 monoclonal antibody not only failed to exert anti-tumor effects, but instead stimulated tumor growth^26^, in support of our results.

In human breast cancer most cytokines are overexpressed, with patients showing increased serum levels^27^. Accordingly, our findings revealed an association between cats with mammary carcinoma and higher serum levels of two pro-inflammatory cytokines (TNF-α and IL-6), positively correlated with serum CTLA-4 levels, as reported in humans and mice^21,28^. A possible reason for this association may be the activation of certain cell types involved in cancer-associated inflammation, like Tregs. Indeed, our results revealed that most tumor samples scored as FoxP3-positive in interstitial lymphocytes (74%), suggesting Treg tumor infiltration, and strongly correlated with interstitial CTLA-4 expression. Interestingly, a significant proportion of the tumors also scored as FoxP3-positive (31%), showing a nuclear staining pattern, in accordance with that described for human breast cancers^29^.

Finally, our results revealed CTLA-4 expression in the cell membrane and cytoplasm of interstitial lymphocytes, showing a strong negative correlation with serum CTLA-4 levels. This relation might indicate the presence of memory T-lymphocytes in more advanced disease stages, as breast cancer patients are known to have significantly more circulating memory T-lymphocytes and fewer naïve T-lymphocytes, and tumor progression and aggressiveness seem to favor the expansion of the former over the latter^30^. Intracellular stores of CTLA‐4 have been shown to be very low in naïve T-lymphocytes, whilst significant amounts are present in memory T-lymphocytes^31^. Although, the overexpression of CTLA-4 in interstitial lymphocytes has been reported in human breast cancer before^32^, it’s correlation with serum CTLA-4 levels, to our best knowledge, had never been studied.

In conclusion, the present study demonstrated a positive association between serum CTLA-4 levels and two pro-inflammatory cytokine serum levels in cats with mammary carcinoma, with higher serum CTLA-4 levels being associated with less aggressive clinicopathological features. We further identified expression of CTLA-4 and FoxP3 in both interstitial and tumor cells, with a positive association between interstitial expression of CTLA-4 and FoxP3, which was also associated with lower serum CTLA-4 levels. Future investigations including a larger and more diverse patient cohort are required to confirm the results, and evaluation of additional T-lymphocyte immune markers and activation phenotypes of various immune cells (including PD-1 and PD-L1) is required to more fully define immune profiles of feline mammary carcinoma patients. Nevertheless, our findings provide further support for spontaneous feline mammary carcinoma as a model for human breast cancer, that can be used to investigate novel immunotherapies that may benefit both human and feline patients.

## Materials and Methods

### Study population

All animals enrolled in this study had a fully documented history of feline mammary carcinoma and were followed up at the Faculty of Veterinary Medicine Teaching Hospital (HEV) between June 2011, and September 2013. Available historical data included age, clinical stage (TNM), malignancy grade, tumor burden and size, regional lymph node involvement, presence of tumor necrosis, lymphatic vessel invasion, lymphocyte infiltration or cutaneous ulceration and histopathological classification (ER status, PR status, HER-2 status, basal status, Ki67 index) (Table 3). Serum samples from 57 female cats were collected at time of admission and conducted after the written consent of the pet owner, in accordance with the principles and procedures outlined in the NIH Guide for the Care and Use of Animals, aliquoted and stored at −80 °C. For 49 of these animals, matching formalin-fixed and paraffin-embedded tumor tissue sections were evaluated. In parallel, serum samples from twelve healthy cats were used as controls for the quantification of serum CTLA-4, TNF-α and IL-6 levels.

**Table 3:** Clinicopathological characteristics of female cats with mammary carcinoma enrolled in the study (n = 57).

Clinical feature		Number (%)
**Breed**	Crossbred	40 (70)
	Persian	7 (12)
	Siamese	7 (12)
	NFC	2 (4)
	Russian blue	1(2)
**Age (years)**	<8	4 (7)
	8–12	31 (54)
	>12	22 (39)
**Stage**	I	15 (26)
	II	7 (12)
	III	31 (54)
	IV	4 (7)
**Grade**	1	3 (5)
	2	8 (14)
	3	46 (81)
**Burden**	Single tumor	21 (37)
	Multiple tumors	36 (63)
**Size (cm)**	<2	20 (35)
	2–3	20 (35)
	>3	17 (30)
**LN status**	Negative	35 (62)
	Positive	18 (32)
	ND	4 (7)
**LVI**	No LVI	50 (88)
	LVI	7 (12)
**LI**	No LI	16 (28)
	LI	39 (68)
	ND	2 (4)
**Necrosis**	No necrosis	15 (26)
	Necrosis	42 (74)
**Ulceration**	No ulceration	50 (88)
	Ulceration	7 (12)
**Ki67 index**	Low (<14%)	18 (32)
	High (>14%)	38 (66)
	ND	1 (2)
**ER status**	Negative	39 (68)
	Positive	18 (32)
**PR status**	Negative	30 (53)
	Positive	27 (47)
**HER-2 status**	Negative	45 (79)
	Positive	12 (21)
**TN status**	Non-TN	42 (74)
	TN	15 (26)
**Basal status**	Non-basal	48 (84)
	Basal like	8 (14)
	ND	1 (2)

LN – Lymph Node; LVI – Lymphatic Vessel Invasion; LI – Lymphocyte Infiltration; ER – Estrogen Receptor; PR – Progesterone Receptor; HER – Epidermal Growth Factor Receptor; TN – Triple Negative; ND – Not Determined.

### Measurement of CTLA-4 and cytokine serum levels

Serum samples were kept frozen at −80 °C and thawed shortly before determination of CTLA-4, TNF-α and IL-6 serum levels. Commercially available immunoassay kits from R&D Systems (R&D Systems, Minneapolis, MN, USA) were used according to the manufacturers’ instructions. CTLA-4 levels were determined with the Mouse CTLA-4 DuoSet ELISA immunoassay kit (code DY476); TNF-α levels were determined with the Feline TNF-α DuoSet ELISA immunoassay kit (code DY2586); and IL-6 levels were determined with the Feline IL-6 DuoSet ELISA immunoassay kit (code DY2305). All kit components were stored at 4 °C. A seven-point standard curve was prepared for each assay by making serial dilutions from a stock of recombinant mouse CTLA-4, feline TNF-α and feline IL-6 provided in the kits. The immunoassays used a solid-phase sandwich enzyme-linked immunosorbent assay (ELISA) technique. Briefly, 96-well microplates were prepared by adding Capture Antibody to each well and incubated overnight. To prevent nonspecific binding the plates were treated with 1% BSA in PBS for 1 h. Afterwards, diluted serum samples were added to each well and incubated for 2 h. Then, the plates were incubated with Biotinylated Detection Antibody for 2 h and Streptavidin Conjugated to Horseradish Peroxidase (HRP) for 20 min. All incubation steps were performed at room temperature and between each step the plates were washed with Wash Buffer (code 895003; R&D) to remove unbound molecules. After the last wash, a substrate solution prepared by mixing equal volumes of TMB (code 895001; R&D) and H_2_O_2_ (code 895000; R&D) was added and the plates were incubated for 20 min. Following color development, sulfuric acid (code 895926; R&D) was added to stop the reaction. The optical density was determined using a FLUOstar OPTIMA microplate reader from BMG Labtech, set to 450 nm. To correct for optical imperfections in the plate a second reading was performed at 570 nm and readings were subtracted from the readings at 450 nm. The data were linearized by plotting the log of the mean absorbance against the log of the concentration using Microsoft Excel version 1904 for Windows (Microsoft Corporation, Redmond, WA, USA). Serum CTLA-4, TNF-α and IL-6 concentrations were determined using the curve fit equation (*y* = *mx + c*) generated. The correlation coefficient between the fitted data and the actual data was greater than 0.99 for all assays.

### Immunohistochemical (IHC) staining

The paraffin-embedded tumor tissue blocks were cut into 3 μm thick sections and transferred to glass slides (SuperFrost Plus, Menzel-Gläser), dried at room temperature, baked in a heated chamber for 1 h at 64 °C, and then left overnight at room temperature. Deparaffinization and antigen retrieval were performed in a PT Link (Dako Denmark A/S) at 97 °C for 20 min, with low pH EnVision FLEX target retrieval solution (code DM829; Dako). Immunohistochemical staining was performed using the Novolink Max Polymer Detection System (Leica Biosystems). The samples were consecutively treated with: peroxidase block (code RE7157) for 20 min; protein block (code RE7158) for 10 min; the optimally diluted primary antibodies for 30 min (Table 4); the post-primary reagent (code RE7159) for 30 min; and finally with the Novolink polymer (code RE7161) for 30 min. The slides were washed between all incubation steps 2 × 5 min in phosphate buffered saline pH 7.4 (PBS). Afterwards, sections were stained with DAB chromogen (code 7162) for 5 min, and nuclei were counterstained with hematoxylin. Slides were dehydrated in an ethanol gradient, mounted with Entellan mounting medium and stored for later observation. Sections of feline lymph node specimens with confirmed high expression of the target molecules served as positive control. Sections of healthy mammary tissue were used as negative control.

**Table 4:** Primary antibodies and their conditions of use.

Antigen	Source clone (code)	Dilution
Synthetic peptide corresponding to human CTLA-4 (internal sequence)	Rabbit monoclonal (Abcam SP355)	1:100 (30′)
FoxP3 fusion protein	Mouse monoclonal (Abcam 236A/E7)	1:100 (30′)

### Interpretation of IHC staining

Two of the authors (AU and JC) evaluated the scores for immunohistochemistry (IHC) grading and diagnostic accuracy. For CTLA-4 IHC staining evaluation, the results were recorded based on the intensity of the staining reaction on the cytoplasm, as well as the estimated percentage of positive interstitial lymphocytes. Interstitial lymphocytes were identified by morphology under a microscope after staining. The scores of percentages of positive cells were recorded as: 0 (<1%), 1 (1–5%), 2 (6–30%) or 3 (>30%). The scores of intensities of positive cells were recorded as: 0 (negative), 1+ (weak), 2+ (moderate), or 3+ (strong) (Fig. 3). The percentage of positive cells and intensity scores were then multiplied to obtain a final IHC score^32^. The FoxP3 expression in interstitial lymphocytes was evaluated in a similar way.

### Statistical analysis

Statistical analysis was carried out using GraphPad Prism version 8.11 for Windows (GraphPad Software, La Jolla, CA, USA). The values p < 0.05 (*), p < 0.01 (**) and p < 0.001 (***) were considered statistically significant. Differences between serum CTLA-4 and cytokine levels in healthy and diseased animals were assessed using the Mann-Whitney U test. The association between serum CTLA-4 levels and various clinicopathological criteria were analyzed by the Kruskal-Wallis test. To control the family wise error rate we adjusted the significance level as α = α/*m* where *m* is the number of simultaneously tested hypotheses^33^. Pearson correlation was used to assess correlations between CTLA-4 and TNF-α/IL-6 serum levels. Cats were divided into positive- and negative-expression groups for lymphocyte CTLA-4 and FoxP3 expression, IHC scores of 0 and 1 were defined as negative and scores >1 as positive. These definitions accounted for the median scores and minimized the difference between the number of animals classified as negative and those classified as positive. Correlations between CTLA-4 serum levels and lymphocyte CTLA-4 and FoxP3 expressions were assessed using the Spearman rank and Mann-Whitney U tests.
